# Genomes of sequence type 121 *Listeria monocytogenes* strains harbor highly conserved plasmids and prophages

**DOI:** 10.3389/fmicb.2015.00380

**Published:** 2015-04-28

**Authors:** Stephan Schmitz-Esser, Anneliese Müller, Beatrix Stessl, Martin Wagner

**Affiliations:** Department for Farm Animals and Veterinary Public Health, Institute for Milk Hygiene, University of Veterinary Medicine ViennaVienna, Austria

**Keywords:** *Listeria monocytogenes*, sequence type 121, persistence, genome, plasmid, prophage

## Abstract

The food-borne pathogen *Listeria (L.) monocytogenes* is often found in food production environments. Thus, controlling the occurrence of *L. monocytogenes* in food production is a great challenge for food safety. Among a great diversity of *L. monocytogenes* strains from food production, particularly strains belonging to sequence type (ST)121 are prevalent. The molecular reasons for the abundance of ST121 strains are however currently unknown. We therefore determined the genome sequences of three *L. monocytogenes* ST121 strains: 6179 and 4423, which persisted for up to 8 years in food production plants in Ireland and Austria, and of the strain 3253 and compared them with available *L. monocytogenes* ST121 genomes. Our results show that the ST121 genomes are highly similar to each other and show a tremendously high degree of conservation among some of their prophages and particularly among their plasmids. This remarkably high level of conservation among prophages and plasmids suggests that strong selective pressure is acting on them. We thus hypothesize that plasmids and prophages are providing important adaptations for survival in food production environments. In addition, the ST121 genomes share common adaptations which might be related to their persistence in food production environments such as the presence of Tn*6188*, a transposon responsible for increased tolerance against quaternary ammonium compounds, a yet undescribed insertion harboring recombination hotspot (RHS) repeat proteins, which are most likely involved in competition against other bacteria, and presence of homologs of the *L. innocua* genes *lin0464* and *lin0465*.

## Introduction

The facultative intracellular pathogen *Listeria monocytogenes* is responsible for listeriosis, a rare but severe disease in humans and animals, which is acquired primarily through the consumption of contaminated food; particularly “ready-to-eat food” is of high risk (Allerberger and Wagner, 2010; Eurosurveillance Editorial, 2012). *L. monocytogenes* can survive and grow in multiple natural and man-made habitats, such as soil, marine and fresh water, vegetation, sewage, food processing plants, farm environments, domestic and wild animals (Sauders and Wiedmann, 2007; Ferreira et al., 2014); therefore, controlling *L. monocytogenes* in food processing environments is a considerable challenge. Long-term survival—also called persistence—for months or even years of various *L. monocytogenes* strains in food production environments has been described by many studies, for reviews see: (Carpentier and Cerf, 2011; Ferreira et al., 2014; Larsen et al., 2014). Persistence describes the repeated occurrence of genetically indistinguishable (determined e.g., by pulsed-field gel electrophoresis or ribotyping) *L. monocytogenes* strains in the same food production plant over a long time period. However, only relatively few studies have focused on the molecular mechanisms of persistence (Carpentier and Cerf, 2011; Ferreira et al., 2014). So far, only two studies have analyzed genome sequences of persistent *L. monocytogenes* strains: one study described the genome of *L. monocytogenes* strain J2818 [sequence type (ST) 11, serovar 1/2a], which persisted for 12 years in a food processing plant in the US (Orsi et al., 2008). Another more recent study determined the genome sequences of two persistent ST121 (serovar 1/2a) *L. monocytogenes* strains isolated from two different fish processing plants in Denmark (Holch et al., 2013). These two studies revealed that prophage diversification is an important driver of *L. monocytogenes* evolution and suggested that specific genetic determinants may enable long-term persistence in food processing environments. Two models explaining persistence have been proposed (Ferreira et al., 2014): According to the first model, certain *L. monocytogenes* strains have unique phenotypic and genotypic characteristics facilitating long-term survival in food processing environments. The other model states that persistence is largely a random process and that most *L. monocytogenes* strains can establish persistence if present in an appropriate niche at an appropriate time (Ferreira et al., 2014). Particularly *L. monocytogenes* strains of ST121 are often found in food production environments (Ragon et al., 2008; Parisi et al., 2010; Chenal-Francisque et al., 2011; Hein et al., 2011; Holch et al., 2013; Kastbjerg et al., 2014; Martin et al., 2014; Stessl et al., 2014; Wang et al., 2015). However, the molecular mechanisms underlying the phenomenon of persistence are currently unknown. As persistent *L. monocytogenes* strains in food processing environments greatly increase the risk of (re)contamination of food products and therefore represent a big challenge for food safety, we analyzed the genome sequences of three ST121 *L. monocytogenes* isolates and compared them with available *L. monocytogenes* ST121 genomes to identify common and unique genetic traits among ST121 genomes with a particular focus on persistence.

## Materials and methods

### Bacterial strains used for genome sequencing

The following three *L. monocytogenes strains* were potentially persisting in three food processing facilities during several years (Table 1). The strains were assigned to genetic lineage II, serovar 1/2a and ST121. *L. monocytogenes* 4423 was isolated primarily from product-associated and cheese samples in 2004 from an Austrian cheese processing plant producing semi-hard and hard cheese (Stessl et al., 2014). *L. monocytogenes* 4423 corresponds to AuB1 and 6179 corresponds to IrlA1 in Stessl et al. (2014). *L. monocytogenes* 6179 originated from an Irish farmhouse-cheesemaking plant and was mainly isolated from cheeses (Fox et al., 2011a,b; Stessl et al., 2014). *L. monocytogenes* 3253 was isolated from Austrian deli-meat products and the corresponding food processing environment (FPE). Details on strains are given in Table 1.

**Table 1:** **Comparison of available *L. monocytogenes* ST121 genomes**.

	**4423**	**6179**	**3253**	**N53-1**	**LM_1880**	**S2_2**	**S2_3**	**S10_1**	**S10_3**
Processing facility	Cheese	Cheese	Deli-meat	Fish	Cheese	Pork meat (plant B)	Pork meat (plant B)	Pork meat (plant A)	Pork meat (plant B)
Country	Austria	Ireland	Austria	Denmark	Italy	Spain	Spain	Spain	Spain
Year of isolation	2004	2000	2014	2002	2012	2010	2010	2008	2010
Occurrence of identical PFGE types/subtypes	1997–2004	2000–2008	2013–2015	1996–2002	n.a.	n.a.	n.a.	2007–2009	n.a.
Source^b^	PA (25), P (22)	P (15), FPE (3)	P (57), PA (37), FPE (9)	n.a.	n.a.	n.a.	n.a.	n.a.	n.a.
Assembly size	3.06 Mbp^a^	3.07 Mbp^a^	3.06 Mbp^a^	3.09 Mbp^a^	3.04 Mbp^a^	3.11 Mbp^a^	3.08 Mbp^a^	3.01 Mbp^a^	3.11 Mbp^a^
Genetic lineage, serovar	II, 1/2a	II, 1/2a	II, 1/2a	II, 1/2a	II, 1/2a	II, 1/2a	II, 1/2a	II, 1/2a	II, 1/2a
No. of prophages	3	3	3	4	3	4	3	1	3
*comK* prophage	−	−	−	−	−	+	+	−	+
tRNA−Arg−CCG prophage	+	+	+	+	+	+	+	+	+
tRNA−Arg−TCT prophage	+	+	+	+	+	+	+	−	+
*lin0464/0465* homologs	+	+	+	+	+	+	+	+	+
Tn*6188*	+	+	+	+	+	+	+	+	+
*Bcr*ABC	−	−	−	−	−	−	−	−	−
truncated *inl*A	+	+	+	+	+	+	+	+	+
Insertion of RHS protein between *lmo2753−lmo2754* homolog*s*	+	+	+	+	+	+	+	+	+
Plasmid size (bp)	62,207	62,206	63,152^a^	60,982^a^	61,066^a^	61,124^a^	61,210^a^	62,492^a^	61,210^a^
References	Stessl et al., 2014	Fox et al., 2011a; Stessl et al., 2014	this study	Holch et al., 2013; Kastbjerg et al., 2014	Chiara et al., 2014	Lopez-Alonso et al., 2015	Lopez-Alonso et al., 2015	Lopez-Alonso et al., 2015	Lopez-Alonso et al., 2015

a *Genomes are not closed; n.a., not available*.

b *P, product; PA, product associated; FPE, food processing environment. Numbers in parentheses indicate the number of isolations of strains with the same PFGE subtype*.

### DNA isolation, genome sequencing and genome analyses

*L. monocytogenes* strains were cultivated under aerobic conditions at 37°C in brain heart infusion broth (BHI, Merck; with 125 rpm shaking), harvested by centrifugation, the resulting pellet was used for DNA isolation using the QIAGEN genomic-tip columns and buffers according to the recommendations of the manufacturer. For 6179 and 4423 genome sequencing was performed using an Illumina GAII genome analyzer available at the University of Veterinary Medicine Vienna. Sequencing was performed using paired-end sequencing technology and 100 bp read-length using Illumina standard protocols. For 3253 genome sequencing was performed with Illumina MiSeq sequencing technology using 300 bp read length and paired-end sequencing (Microsynth, Balgach, Switzerland). Four (4423) and three (6179 and 3253) million reads were used for a *de novo* assembly using SeqManNGen (DNASTAR). The average coverage was 145 × for *L. monocytogenes* 4423, 98 × for *L. monocytogenes* 6179, and 205 × for 3253. This assembly resulted in 35 contigs with a size >500 bp for 4423, 32 contigs for 6179, and 12 contigs for 3253. The contigs were aligned to the *L. monocytogenes* EGDe genome using the “move contigs” option in MAUVE (Darling et al., 2010) and used for initial genome analyses. PCR and Sanger sequencing was performed to close remaining gaps—this resulted in one contig for 6179 and 12 contigs for 4423. Automatic genome analysis and annotation of the genomes was done using the RAST server (http://rast.nmpdr.org/) (Aziz et al., 2008; Overbeek et al., 2014) and the MicroScope platform (https://www.genoscope.cns.fr/agc/microscope/home/) (Vallenet et al., 2013). Genome comparisons and determination of homologous proteins were done with BlastP, BlastN, and tBlastN (Camacho et al., 2009). Similar to a previous study (Kuenne et al., 2013) we used a similarity cut-off of 60% amino acid identity and 80% coverage for identification of homologous proteins. Alignments of genomes, prophages and plasmids were done with MAUVE (Darling et al., 2010). Multilocus sequence typing (MLST) of the sequenced strains was performed with the MLST tool available on the Center for Genomic Epidemiology website [https://cge.cbs.dtu.dk/services/MLST/ (Larsen et al., 2012)]. For comparison other currently available ST121 genomes: the genome sequences of LM_1880 (GenBank accession number AZIZ00000000), a strain isolated from cheese in Italy—for which no information regarding possible persistence is currently available—(Chiara et al., 2014) and N53-1 (GenBank accession number AXDU01000000), a persistent *L. monocytogenes* strain isolated from a fish production plant in Denmark (Holch et al., 2013; Kastbjerg et al., 2014) and S2_2, S2_3, S10_1, and S10_3 (GenBank accession numbers: JWHJ01000000, JWHK01000000; JWHG01000000, and JWHH01000000), isolated from pork industry in Spain (Ortiz et al., 2010, 2014; Lopez-Alonso et al., 2015) were downloaded from GenBank and loaded onto the RAST server. In the study by Holch and coworkers (Holch et al., 2013) it was stated that the strains La111 and N53-1 were almost identical (no SNPs present), we therefore used only N53-1 for our comparisons. CRISPR regions were analyzed with CRISPRFinder [http://crispr.u-psud.fr/Server/CRISPRfinder.php (Grissa et al., 2007)]. Phylogenetic analyses of plasmid replication initiation protein amino acid sequences was performed with MEGA 6.0 using maximum likelihood based phylogenetic inference and the JTT amino acid substitution model with 1000x bootstrapping (Tamura et al., 2013).

### Accession numbers

The genome and plasmid sequences have been deposited in the EMBL European nucleotide archive under accession numbers HG813249 and HG813250 for *L. monocytogenes* 6179 and CBXR010000001 to CBXR010000012 for *L. monocytogenes* 4423. The *L. monocytogenes* 3253 whole genome shotgun project has been deposited at DDBJ/EMBL/GenBank under the accession JYJO00000000. The version described in this paper is version JYJO01000000.

## Results and discussion

Recently, two *L. monocytogenes* sequence type (ST) 121 strains (4423 and 6179) were found to be potentially persistent in European cheese processing facilities (Fox et al., 2011a,b; Stessl et al., 2014). The genetically highly similar (but not identical) *L. monocytogenes* strains 4423 and 6179 were recurrently isolated during a timeframe of seven and 8 years (Table 1). Interestingly, a high abundance of *L. monocytogenes* ST121 in food and persistence in FPE was also observed by other authors (Parisi et al., 2010; Chenal-Francisque et al., 2011; Holch et al., 2013; Althaus et al., 2014; Martin et al., 2014; Ortiz et al., 2014; Wang et al., 2015). Currently, 69 *L. monocytogenes* ST121 are available in the Institute Pasteur MLST database with a considerable increased isolation history from 2009 to 2014 (http://www.pasteur.fr/recherche/genopole/PF8/mlst/Lmono.html; accessed on: 19.02.2015).

To get more insight into the genetic features involved in *L. monocytogenes* ST121 persistence in FPE, the three ST121 strains 4423, 6179, and 3253 were selected for whole genome sequencing and compared with available *L. monocytogenes* ST121 genomes (*n* = 6).

Genome sequencing followed by gap closing using PCR resulted in one contig for *L. monocytogenes* 6179 (Table 2), however we were not able to close the genome. The remaining gap probably comprised two consecutive rRNA operons as in the homologous region in *L. monocytogenes* EGDe. For *L. monocytogenes* 4423 and 3253, 12 contigs remained (Table 2). The genomes display typical features of *L. monocytogenes* genomes such as assembly sizes between 3.00 and 3.01 Mbp and a genomic G+C content of 37.9%, which are in the range found for most *Listeria* genomes (den Bakker et al., 2010, 2013; Kuenne et al., 2013). In addition, all strains harbor a plasmid (pLM6179, pLM4423, and pLM3253) with sizes of 62.2 (6179 and 4423) and 63.1 kbp (3253) with G+C content of 36.5%. Details on the genomes and plasmids are shown in Table 2.

**Table 2:** **General features of the *L. monocytogenes* 6179, 4423, and 3253 genomes and plasmids determined in this study**.

	**3253**	**4423**	**6179**	**pLM3253**	**pLM4423**	**pLM6179**
Assembly size (bp)	3,001,169^a^	3,000,849^a^	3,010,620^a^	63,152^a^	62,207	62,206
No. of contigs	11	11	1	1	1	1
G+C content (%)	37.9	37.9	37.9	36.5	36.5	36.5
No. of predicted coding sequences (CDS)	2990	3054	3063	62	62	61
Average length of CDS	890	889	886	892	892	902
Coding density (%)	89.4	89.5	89.4	88.8	88.8	88.3
No. of rRNA operons	5^b^	6	4^b^	–	–	–
No. of tRNA genes	63	61	49	–	–	–
No. of prophages	3	3	3	–	–	–

a *Genomes are not closed*.

b *The number of rRNA operons could not be determined due to remaining gaps in the assembly*.

**Figure 1:**
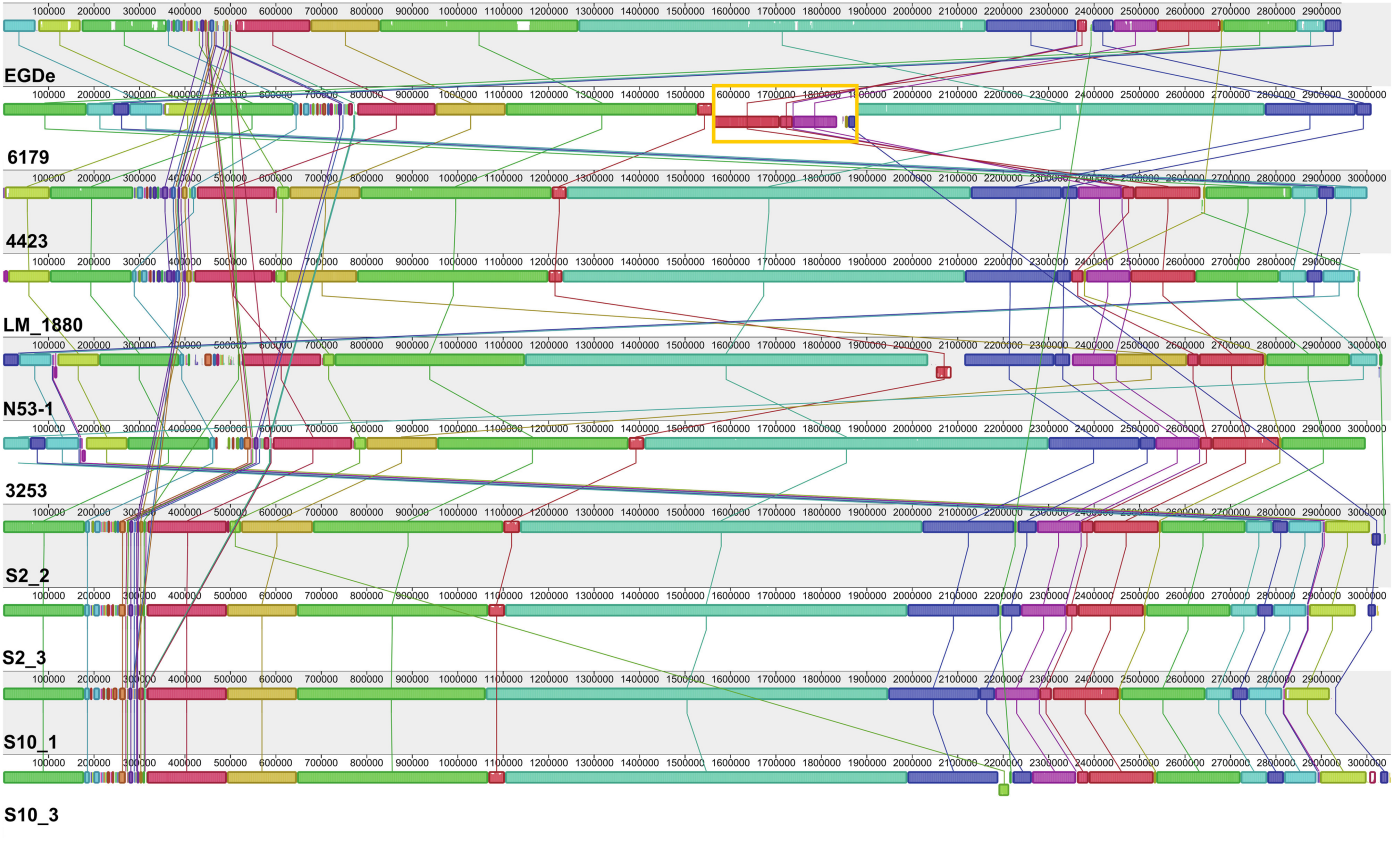
**Alignment of *L. monocytogenes* ST121 genomes**. The genomes of *L. monocytogenes* EGDe (ST35), 6179, 4423; N53-1, 3253, LM_1880, S2_2, S2_3, S10_1, and S10_3 were aligned using Mauve (Darling et al., 2010). Homologous regions are shown in the same color. The height of the similarity profile within each block corresponds to the average level of conservation in that region of the genomes. The rearrangement in the *L. monocytogenes* 6179 genome is highlighted in orange.

Overall, all the sequenced ST121 genomes are highly similar to each other and also highly similar to the *L. monocytogenes* EGDe genome (Figure 1, Table 1); however, one 358 kbp inversion in the 6179 genome between the EGDe homologs *lmo1240* (*LM6179_1548*) and *lmo1241* (*LM6179_1975*) representing homologs of the EDGe genes *lmo2589* to *lmo2366* including also all three 6179 prophages, was found. This rearrangement most likely explains the slightly different PFGE patterns of 4423 and 6179 reported recently (Stessl et al., 2014). The average nucleotide and amino acid identity between the nine ST121 strains is 99.8 and 99.9%, respectively.

The ST121 genomes harbor between one and four prophages (Table 1). In 6179, 4423, and 3253, one prophage is inserted downstream of the tRNA Arg-TCT, another prophage downstream of tRNA Arg-CCG. In 6179, the third phage is inserted downstream of the tRNA Thr-GGT and in 4423 and 3253 upstream of the tRNA Ser-CGA. Prophages or phage remnants integrated into the *comK* gene are found in three of the nine ST121 genomes. Prophages integrated into *comK* have been suggested to be important for the adaptation of *L. monocytogenes* to food environments (Verghese et al., 2011).

The phages at tRNA Ser-CGA in 4423 and 3253 and tRNA Thr-CGT in 6179 are specific for the respective strains (but show highest similarity to other *Listeria* phages). Interestingly, in 6179, 4423, and 3253, the phages at tRNA Arg-CCG are identical and the phages inserted at tRNA Arg-TCT show more than 96.5% nucleotide sequence identity to each other (Supplementary Figures 1, 2). Highly similar tRNA Arg-CCG (99.8 to 100% nucleotide identity) and tRNA Arg-TCT (95.4 to 100% nucleotide identity) prophage contigs are also present in LM_1880, N53-1, S2_2, S2_3, S10_1, and S10_3 (Supplementary Figures 1, 2). The similarity of the ST121 tRNA Arg-TCT and tRNA Arg-CCG prophages to other described *Listeria* phages is considerably lower (Supplementary Figures 1, 2). This high degree of similarity of prophages—particularly of tRNA-Arg-CCG prophages—in *L. monocytogenes* ST121 strains isolated independently from food processing plants from five different countries (Austria, Ireland, Denmark, Italy, and Spain) in different years is striking, particularly keeping in mind that prophages have been shown to be important drivers of short-term genome evolution in *L. monocytogenes* (Orsi et al., 2008; Gilmour et al., 2010; Verghese et al., 2011) and are considered to be the major source of diversity within the genus *Listeria* (Kuenne et al., 2013). Two recent studies performed comparative genome analyses of *Listeria* bacteriophages also found regions showing high conservation between some phages, but to a much lesser degree as found in our study (Dorscht et al., 2009; Denes et al., 2014). The high degree of similarity particularly of the tRNA Arg-CCG prophages identified here might thus be a result of adaptation of ST121 strains to similar niches in food production environments. It has been shown that prophages can provide increased growth under nutrient limitation (Edlin et al., 1977), increased biofilm formation (Wang et al., 2010; Verghese et al., 2011; Fortier and Sekulovic, 2013) and can be beneficial for withstanding osmotic, oxidative, and acid stress (Wang et al., 2010). In a similar way, the presence and high conservation of *L. monocytogenes* ST121 prophages might thus be advantageous for survival under stress conditions, which they are faced with in food production environments.

All analyzed ST121 genomes encode a typical *L. monocytogenes* pathogenicity island and truncated internalin A (inlA) genes, a feature often found in ready-to-eat food and food production environment *L. monocytogenes* isolates (Nightingale et al., 2008; Van Stelten et al., 2010). The truncated InlA proteins have a predicted length of 492 amino acids and belong to mutation type 6 (Nightingale et al., 2008; Van Stelten et al., 2010). Overall, the genomic organization of the *inlAB* locus in ST121 strains is similar to *L. monocytogenes* EGDe (Supplementary Figure 3). Interestingly, the *lmo0435* homolog BapL, a putative peptidoglycan bound protein involved in biofilm formation, but not essential (Jordan et al., 2008), is truncated in ST121 strains (Supplementary Figure 3). All ST121 strains encode a highly similar set of internalins and internalin-like proteins (Supplementary Tables 1, 2). Using a set of virulence-associated genes based on a study by den Bakker et al. (2010), we performed BlastP and tBlastN searches: all virulence genes present in *L. monocytogenes* EGDe except homologs of *lmo2026* (an internalin-like protein) are present in the ST121 genomes (data not shown).

*Listeria* genomes are highly syntenic and horizontal gene transfer into *Listeria* genomes occurs mostly in the accessory genome consisting mainly of prophages, transposons and so-called hypervariable hotspots (den Bakker et al., 2010, 2013; Kuenne et al., 2013). We thus compared the regions of the hypervariable hotspots in the ST121 genomes to identify possible differences and found the same gene content in all hypervariable hotspots; only in LM_1880, hypervariable hotspot 7 (*lmo0458* to *lmo0480*) seems to be part of a rearrangement, however also here the gene content is identical to the other ST121 genomes (data not shown). One mechanism for protection against foreign DNA are restriction modification systems. The ST121 genomes encode a lmoJ2 type II restriction modification system locus inserted into hypervariable hotspot 4 (*lmo0301* to *lmo0314*); LmoJ2 has been shown to be involved in phage resistance (Lee et al., 2012). In addition, all ST121 genomes encode two recently described CRISPR loci: a type I CRISPR system inserted between *lmo0517* and *lmo0518* homologs, and a type II CRISPR system inserted between *lmo2591* and *lmo2595* as described previously in other *L. monocytogenes* genomes (Kuenne et al., 2013; Sesto et al., 2014). We analyzed the CRISPR regions in the ST121 genomes: overall, the CRISPR regions were identical, with the exception of a single 14 bp insertion in CRISPR locus 1 in 6179 and the presence of only 21 spacers in 4423 compared to other complete CRISPR 2 loci in ST121 genomes (Supplementary Table 3); however, in 6179, LM_1880, and N53-1 CRISPR 2 loci were located at the end of contigs and thus not completely assembled.

*L. monocytogenes* encounters various stress conditions in food and food production environments. We therefore analyzed the ST121 genomes with respect to genes possibly involved in stress tolerance. Previously, the so-called stress survival islet 1 (SSI-1) has been characterized in *L. monocytogenes* and was shown to confer increased tolerance toward acidic and salt stress (Ryan et al., 2010). Similar to the results of a recent study, all ST121 genomes harbor homologs of the *L. innocua* genes *lin0464* and *lin0465* at the same genomic locus (Hein et al., 2011). However, their function is currently unknown.

**Figure 2:**
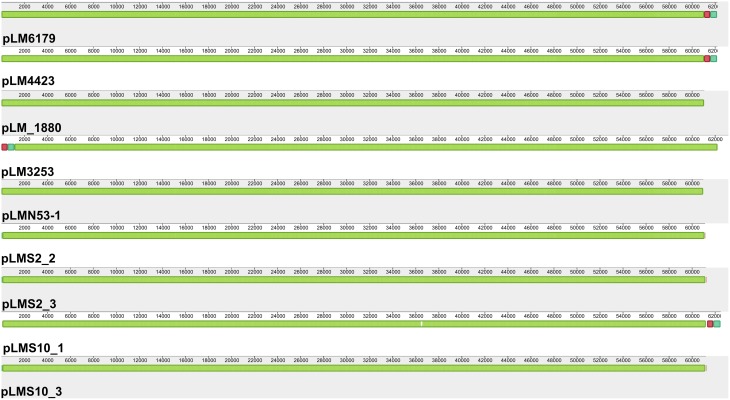
**Alignment of *L. monocytogenes* ST121 plasmids**. The plasmids pLM6179, pLM4423, pLMN53-1, pLM_1880, pLM3253, pLMS2_2, pLMS2_3, pLMS10_1, and pLMS10_3 were aligned using Mauve (Darling et al., 2010). Homologous regions are shown in the same color. The height of the similarity profile within each block corresponds to the average level of conservation in that region of the plasmids.

Plasmids also often confer increased stress tolerance. The pLM4423 and pLM6179 plasmid genome sequences were closed by PCR and have a size of 62.2 kbp (Table 2). They encode typical features found in many *Listeria* plasmids such as determinants for plasmid maintenance, replication and possible transfer as well as the CadAC Cadmium resistance transposon Tn*5422* (Lebrun et al., 1994a,b; Kuenne et al., 2010) and also ClpL homologs, which are members of the HSP100 subgroup of heatshock proteins. ClpL proteins were found in some *Listeria* plasmids before (Kuenne et al., 2010). Highly similar (approximately 68% amino acid identity) ClpL proteins have been shown to be involved in stress response, virulence under various conditions in *Streptococcus* spp. and *Lactobacillus* spp. and to act as chaperone for the stress response regulator CtsR (Suokko et al., 2005; Kajfasz et al., 2009; Tran et al., 2011; Tao and Biswas, 2013). The plasmid-encoded ClpL proteins thus most likely provide additional stress response potential to ST121 strains. Most of the other predicted proteins of ST121 plasmids have no known function. All currently sequenced ST121 *L. monocytogenes* harbor a plasmid with assembly sizes between 60.9 and 63.1 kbp (Table 1). Based on phylogenetic analyses of plasmid replication initiation proteins, these plasmids belong to group 2 *Listeria* plasmids (Supplementary Figure 4). Strikingly, these plasmids are basically identical (Figure 2, Supplementary Figure 4) and show more than 99.9% nucleotide identity to each other. The only visible differences in the alignments (Figure 2) are due to the fact that only the pLM6179 and pLM4423 plasmid sequences are closed. The similarity to their most similar related plasmids is considerably lower: 99.3% nucleotide identity (coverage: 78%) to pLM5578 from *L. monocytogenes* 08-5578 (ST120) (Gilmour et al., 2010) and 96.6% (coverage: 74%) to pLMR479a from *L. monocytogenes* R479a (ST8) (Supplementary Figure 5). This extremely high degree of conservation between plasmids of strains isolated from different sources, years and countries suggests that strong selective pressure is acting on the ST121 plasmids. This high conservation of ST121 plasmids may thus be the result of niche adaptation. Plasmids are thought to have important ecological functions because they can be found in high abundance in bacterial populations in diverse habitats and encode a wide array of accessory functions which may confer an advantage to their bacterial hosts, compensating for the burden of carrying a plasmid (Heuer et al., 2008). In addition, it has been suggested that not only bacterial taxa but also their plasmids are defined by their respective ecological niches (Brown Kav et al., 2012). High similarity among some *Listeria* plasmid sequences has been observed by previous studies (Canchaya et al., 2010; Kuenne et al., 2010). However, in these cases the regions of high similarity represented either only parts of the plasmid sequences or plasmids such as pLM33 with a size of 32 kbp integrated into other larger plasmids and not—as found here—the whole plasmid genomes. Another possible explanation for the high conservation observed in ST121 plasmids and prophages might be that these mobile genetic elements show a high dispersal rate. Assuming a high dispersal rate of these mobile genetic elements nevertheless strongly suggests an advantage of their presence. In line with this, we could show a high level of transcription and differential expression (more than threefold change) of 75 of the tRNA-Arg-TCT and tRNA-Arg-CCG prophage genes and also of 30 out of 62 plasmid genes in *L. monocytogenes* 6179 under benzethonium chloride challenge (Casey et al., 2014); suggesting an important functional role of these mobile genetic elements at least under the conditions analyzed in this study. In addition, the strong and wide transcription of these genes suggests that negative selection is not acting on these prophages and plasmids.

In food production environments, bacteria are regularly faced with cleaning and disinfectants, thus resistance mechanisms may provide important advantages. Recently, we identified the novel transposon Tn*6188* in *L. monocytogenes* 6179 and 4423, which is involved in increased tolerance to various quaternary ammonium compounds such as benzalkonium chloride. We found Tn*6188* in 11% of the analyzed strains, the Tn*6188*-positive strains were primarily of serovar 1/2a (Muller et al., 2013, 2014). Interestingly, identical Tn*6188* copies are present in all ST121 strains analyzed in this study. However, the Tn*6188* copies in N53-1 and LM-1880 were not correctly annotated in these two genomes. In addition, a different benzalkonium chloride resistance mechanism, the bcrABC cassette has been characterized in *L. monocytogenes* H7550 (a serovar 4b strain) and was later found in other *L. monocytogenes* genomes and plasmids (Elhanafi et al., 2010; Dutta et al., 2013). In contrast to Tn*6188*, bcrABC cassettes are absent from the sequenced ST121 genomes.

**Figure 3:**
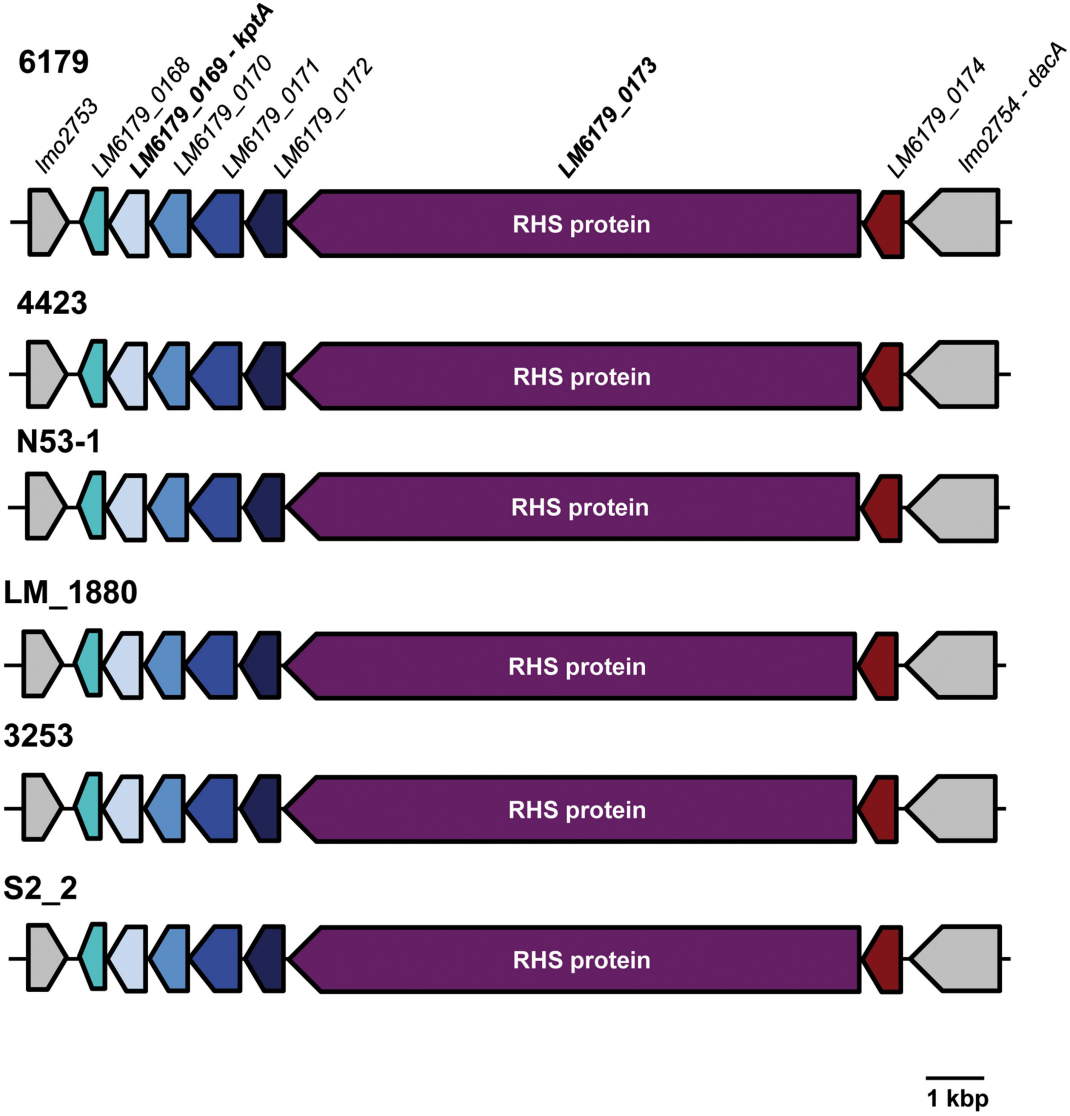
**Organization of the insertion between *lmo2753* and *lmo2754* homologs in *L. monocytogenes* ST121 strains**. Homologous genes are shown in the same color. The inserted region is identical in all analyzed ST121 genomes. For clarity, only one of the Spanish pork industry isolates (S2_2) and only *L. monocytogenes* 6179 locus_tags are shown.

We found a 12.5 kbp insertion in the ST121 genomes between the EGDe *lmo2753* and *lmo2754* homologs. This insertion has a G+C content of 40.0%, which is slightly higher than the average genomic G+C content of *L. monocytogenes* genomes, and encodes—among others—a 3056 amino acid protein harboring 29 rearrangement hotspot (RHS) repeats (PFAM domain: PF05593, locus_tag: LM6179_0173, Figure 3). The RHS proteins and the region surrounding them in ST121 strains are identical. RHS repeat harboring proteins such as RhsAB from *E. coli* or *Dickeya dadantii* (the former *Erwinia chrysanthemi*) or WapA from *Bacillus subtilis* have been shown to be involved in intercellular competition by inhibiting growth of neighboring cells (Koskiniemi et al., 2013). More generally, RHS proteins have been found in many diverse bacteria and been shown or suggested to be involved in cell-cell interactions (Busby et al., 2013; Kwong et al., 2014). Generally, the conservation among RHS proteins is low (Busby et al., 2013). In line with this, the *L. monocytogenes* ST121 RHS proteins show only 25% amino acid identity to WapA from *B. subtilis*, which is responsible for tRNA cleavage resulting in inhibition of cell growth of neighboring cells (Koskiniemi et al., 2013). Upstream of the RHS protein in ST121 strains, a putative RNA 2′-phosphotransferase (KptA) is present (locus_tag: LM6179_0169). The ST121 KptA proteins show 39% amino acid identity to the functionally characterized *E. coli* KptA. Interestingly, RNA 2′-phopsphotransferases have been shown to perform RNA cleavage by a mechanism highly similar to ADP-ribosylation catalyzed by various bacterial toxins (Spinelli et al., 1999). Although not directly shown, most likely, KptA also catalyzes tRNA cleavage (Spinelli et al., 1999). Thus, the putative RNA 2′-phosphotransferases in ST121 strains might fulfill a similar function than the nuclease domain of WapA in *B. subtilis*. In this context it is tempting to speculate that the presence of this insertion might relate to the widespread occurrence of *L. monocytogenes* ST121 strains in food production environments. We hypothesize that the presence of this insertion harboring the RHS protein and the putative RNA 2′-phosphotransferases provides ST121 strains with a means for better competition against other bacteria in food production environments.

Taken together, our results show that *L. monocytogenes* ST121 strains are highly similar to each other and also show an extremely high degree of conservation in some prophage regions and in their plasmids. This high level of conservation suggests that a strong selective pressure is acting on these conserved prophages and plasmids, which are usually among the most variable parts of genetic information in bacteria. It is tempting to speculate that these conserved regions provide ST121 strains with fitness adaptations possibly enhancing survival in food production environments as shown e.g., for other prophages. In addition, we show that *L. monocytogenes* ST121 strains harbor common genetic determinants such as Tn*6188*, or the insertion harboring the RHS proteins, which might also increase their chances of becoming persistent. However, more *L. monocytogenes* ST121 genome sequences, genomes from persistent strains from other sequence types and experimental validation will be needed to confirm our hypotheses. A possible contribution of *L. monocytogenes* ST121 plasmids to survival in food production environments could, e.g., be tested by curing strains from their plasmids or by deleting specific plasmid genes of interest such as the genes encoding the ClpL proteins. Similarly, a contribution of prophages to persistence could be tested by deleting the prophages and monitoring the phenotypic effects on survival under conditions similar to food production environments. Likewise, a possible role of other chromosomally encoded genes in persistence such as the RHS proteins could be analyzed by generating deletion mutants and investigating their effect on survival.

### Conflict of interest statement

The authors declare that the research was conducted in the absence of any commercial or financial relationships that could be construed as a potential conflict of interest.
