# Supplementary material for: Genomes of sequence type 121 Listeria monocytogenes strains harbor highly conserved plasmids and prophages
Source: Front Microbiol. 2015 Apr 28;6:380. doi: 10.3389/fmicb.2015.00380 (PMC4412001; doi:10.3389/fmicb.2015.00380)
Supplement: Supplementary file 1 [file Table1.PDF]

Supplementary Table 1: Presence of internalins in *L. monocytogenes* ST121 genomes.

	4423	6179	N53-1	LM_1880	3253	S2_2	S2_3	S10_1	S10_3
inlC2 *(LMOf8654_0275)*	+	+	+	+	+	+	+	+	+
inlD *(LMOf8654_0276)*	+	-	+	+	+	+	+	+	+
inlE *(lmo0264)*	+	+	+	+	+	+	+	+	+
inlG *(lmo0262)*	-	-	-	-	-	-	-	-	-
inlH *(lmo0263)*	-	-	-	-	-	-	-	-	-
inlI *(lmo0333)*	+	+	+	+	+	+	+	+	+
inlF *(lmo0409)*	-	-	-	-	-	-	-	-	-
inlA *(lmo0433)*	truncated	truncated	truncated	truncated	truncated	truncated	truncated	truncated	truncated
inlB *(lmo0434)*	+	+	+	+	+	+	+	+	+
inlK *(lmo1290)*	+	+	+	+	+	+	+	+	+
inlC *(lmo1786)*	+	+	+	+	+	+	+	+	+
inlJ *(lmo2821)*	+	+	-	+	+	+	+	+	+

The selection of internalins is based on Bjerne et al., Microbes Infect. 2007 (10):1156-66
