# Supplementary material for: Genomes of sequence type 121 Listeria monocytogenes strains harbor highly conserved plasmids and prophages
Source: Front Microbiol. 2015 Apr 28;6:380. doi: 10.3389/fmicb.2015.00380 (PMC4412001; doi:10.3389/fmicb.2015.00380)
Supplement: Supplementary file 2 [file Table2.PDF]

Supplementary Table 2: Presence of internalin-like proteins in *L. monocytogenes* ST121 genomes.

Internalin-like protein	4423	6179	N53-1	LM_1880	3253	S2_2	S2_3	S10_1	S10_3
lmo0171	+	+	+	+	+	+	+	+	+
lmo0327	truncated	truncated	truncated	truncated	truncated	truncated	truncated	truncated	truncated
lmo0331	+	+	+	+	+	+	+	+	+
lmo0514	truncated	truncated	truncated	truncated	truncated	truncated	truncated	truncated	truncated
lmo0549	+	+	+	+	+	+	+	+	+
lmo0610	+	+	+	+	+	+	+	+	+
lmo0732	+	+	+	+	+	+	+	+	+
lmo0801	+	+	-	+	+	+	+	+	+
lmo1136	+	+	+	+	+	+	+	+	+
lmo1289	+	+	+	+	+	+	+	+	+
lmo2026	-	-	-	-	-	-	-	-	-
lmo2027	+	+	+	+	+	+	+	+	+
lmo2396	truncated	truncated	truncated	truncated	truncated	truncated	truncated	truncated	truncated
lmo2445	+	+	+	+	+	+	+	+	+
lmo2470	+	+	+	+	+	+	+	+	+
LMOf6854_0284	+	+	+	+	+	+	+	+	+
LMOf6854_0364	+	+	+	+	+	+	+	+	+
LMOf6854_0833	+	+	+	+	+	+	+	+	+

The selection of internalin-like proteins is based on Bjerne et al., Microbes Infect. 2007 (10):1156-66
