# Supplementary material for: Genomes of sequence type 121 Listeria monocytogenes strains harbor highly conserved plasmids and prophages
Source: Front Microbiol. 2015 Apr 28;6:380. doi: 10.3389/fmicb.2015.00380 (PMC4412001; doi:10.3389/fmicb.2015.00380)
Supplement: Supplementary file 3 [file Table3.PDF]

Supplementary Table 3: CRISPR regions in *Listeria monocytogenes* ST121 genomes

CRISPR_1 (*lmo0517/lmo0518*)									
	**6179**	**4423**	**LM_1880**	**N53-1**	**3253**	**S2_2**	**S2_3**	**S10_1**	**S10_3**
No of spacers	35	35	35	35	35	35	35	35	35
Direct repeat length	29	29	29	29	29	29	29	29	29
Direct repeat sequence	GTTTTAACTACTTATTATGAAATGTAAAT								
CRISPR length	2310	2296	2296	2296	2296	2296	2296	2296	2296
**CRISPR_2 (*lmo2591/lmo2595*)**									
No of spacers	5	21	16	14	22	22	22	22	22
Direct repeat length	36	36	36	36	36	36	36	36	36
Direct repeat sequence	GTTTTAGAGCTATGTTATTTTGAATGCTACCAAAAC								
CRISPR length	365*	1420	1092*	959*	1486	1486	1486	1486	1486

*CRISPR regions at the end of contigs
