# Supplementary material for: Genomes of sequence type 121 Listeria monocytogenes strains harbor highly conserved plasmids and prophages
Source: Front Microbiol. 2015 Apr 28;6:380. doi: 10.3389/fmicb.2015.00380 (PMC4412001; doi:10.3389/fmicb.2015.00380)
Supplement: Supplementary file 4 [file Image1.PDF]

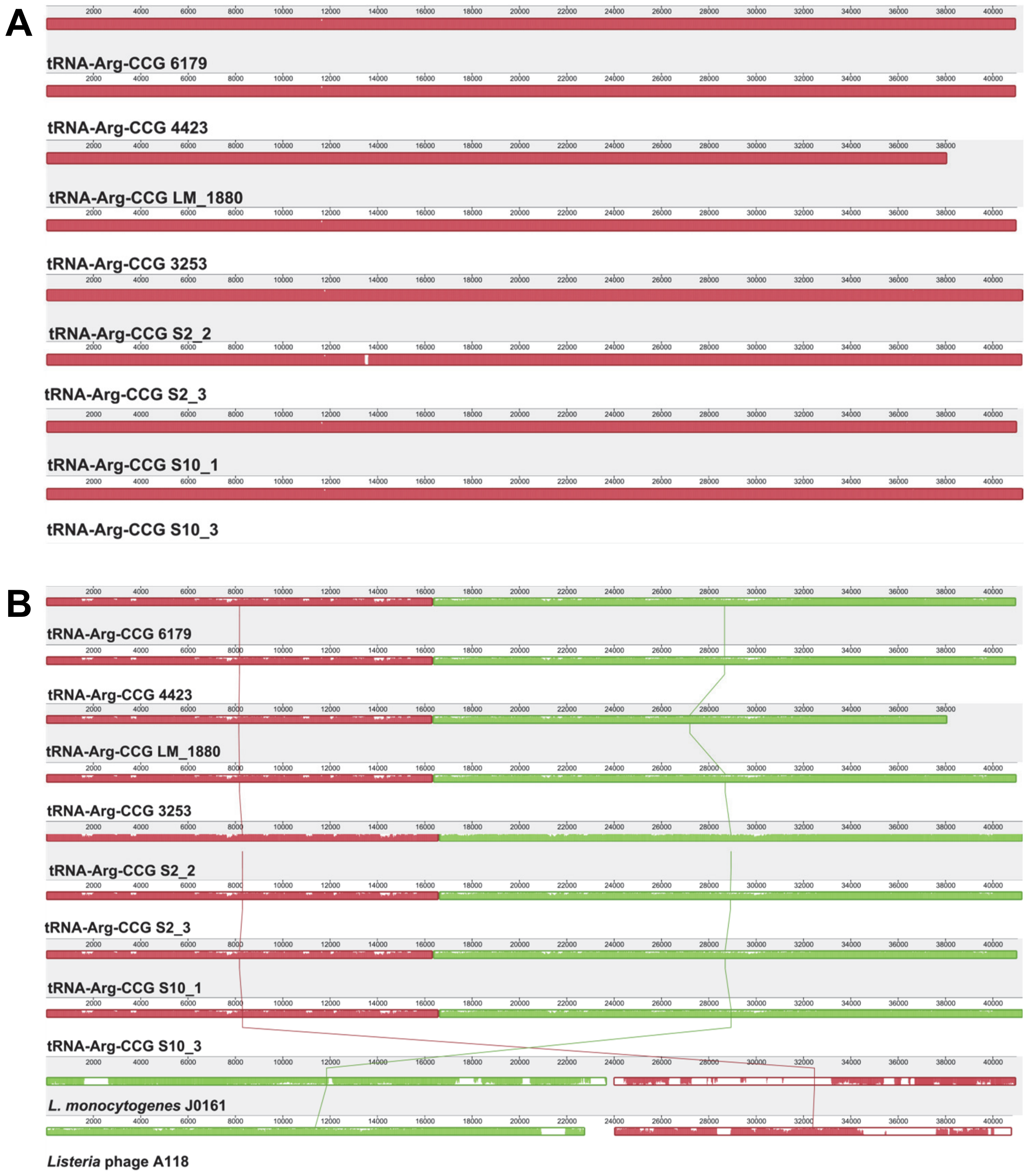

Supplementary Figure 1: DNA-based alignments of prophages from *L. monocytogenes* strains inserted at tRNA Arg-CCG. (**A**) Comparison of *L. monocytogenes* ST121 tRNA-Arg-CCG prophages and (**B**) comparison of *L. monocytogenes* ST121 tRNA-Arg-CCG prophages with related phages from *L. monocytogenes* J0161 (ST11) and with the *Listeria* phage A118. Alignments were created with Mauve (Darling, A.E., et al. (2010) PLoS One 5, e11147). Homologous regions are shown in the same color. The height of the similarity profile within each block corresponds to the average level of conservation.
