# Supplementary material for: Genomes of sequence type 121 Listeria monocytogenes strains harbor highly conserved plasmids and prophages
Source: Front Microbiol. 2015 Apr 28;6:380. doi: 10.3389/fmicb.2015.00380 (PMC4412001; doi:10.3389/fmicb.2015.00380)
Supplement: Supplementary file 7 [file Image4.PDF]

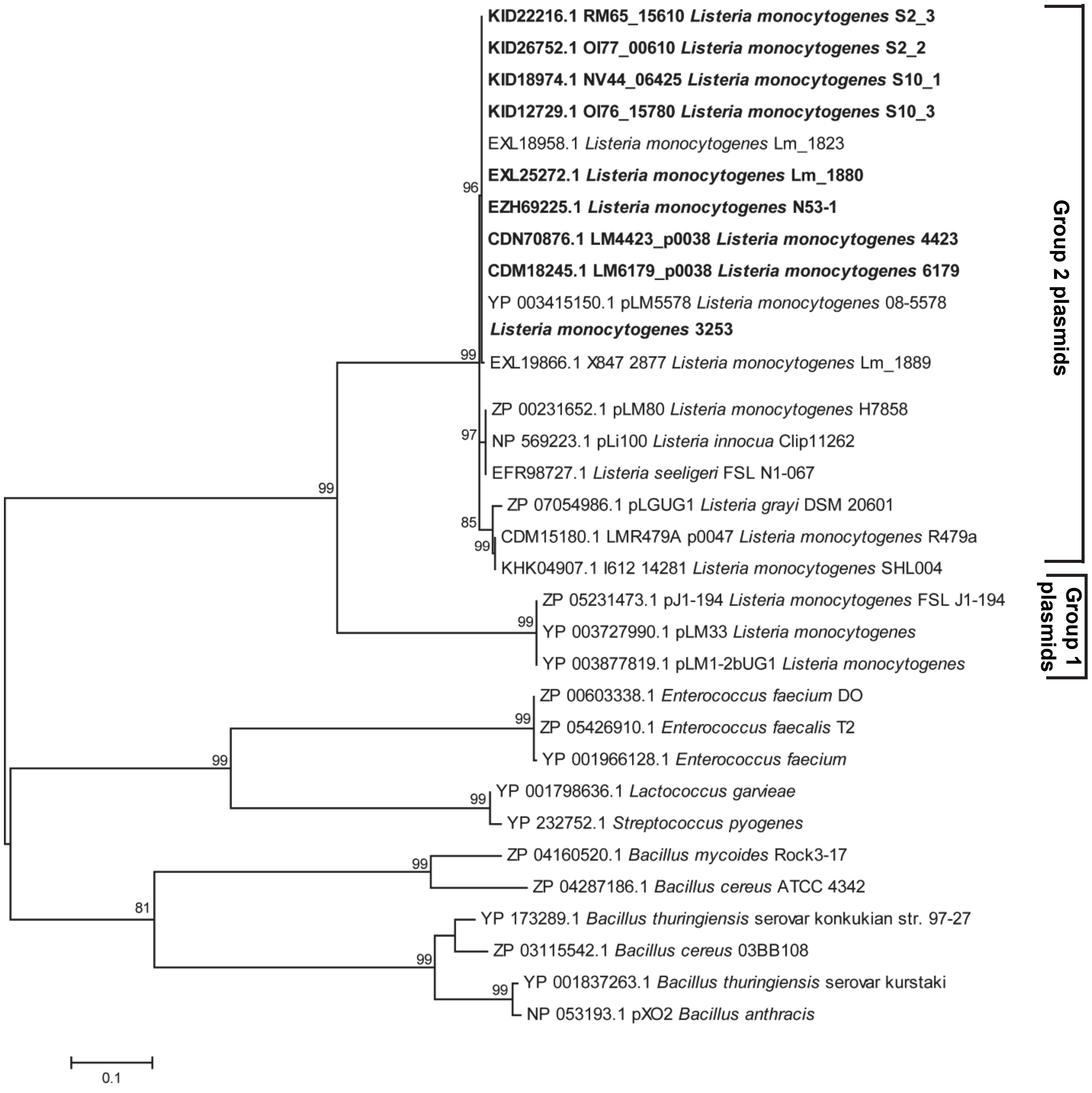


**Supplementary Figure 4. Phylogenetic relationships of *Listeria* plasmids.** The evolutionary history was inferred by using amino acid sequences of RepA replication initiation proteins and the Maximum Likelihood method based on the JTT matrix-based model. The tree is drawn to scale, with branch lengths measured in the number of substitutions per site. Bootstrap values (1000 replications) higher than 90 are indicated at the branches. The analysis involved 32 amino acid sequences. All positions containing gaps and missing data were eliminated. There were a total of 365 positions in the final dataset. Evolutionary analyses were conducted in MEGA6 (Tamura K., et al. 2013 Mol Biol Evol 30: 2725-2729). ST121 sequences are highlighted in boldface.
