# Supplementary material for: Genomes of sequence type 121 Listeria monocytogenes strains harbor highly conserved plasmids and prophages
Source: Front Microbiol. 2015 Apr 28;6:380. doi: 10.3389/fmicb.2015.00380 (PMC4412001; doi:10.3389/fmicb.2015.00380)
Supplement: Supplementary file 8 [file Image5.PDF]

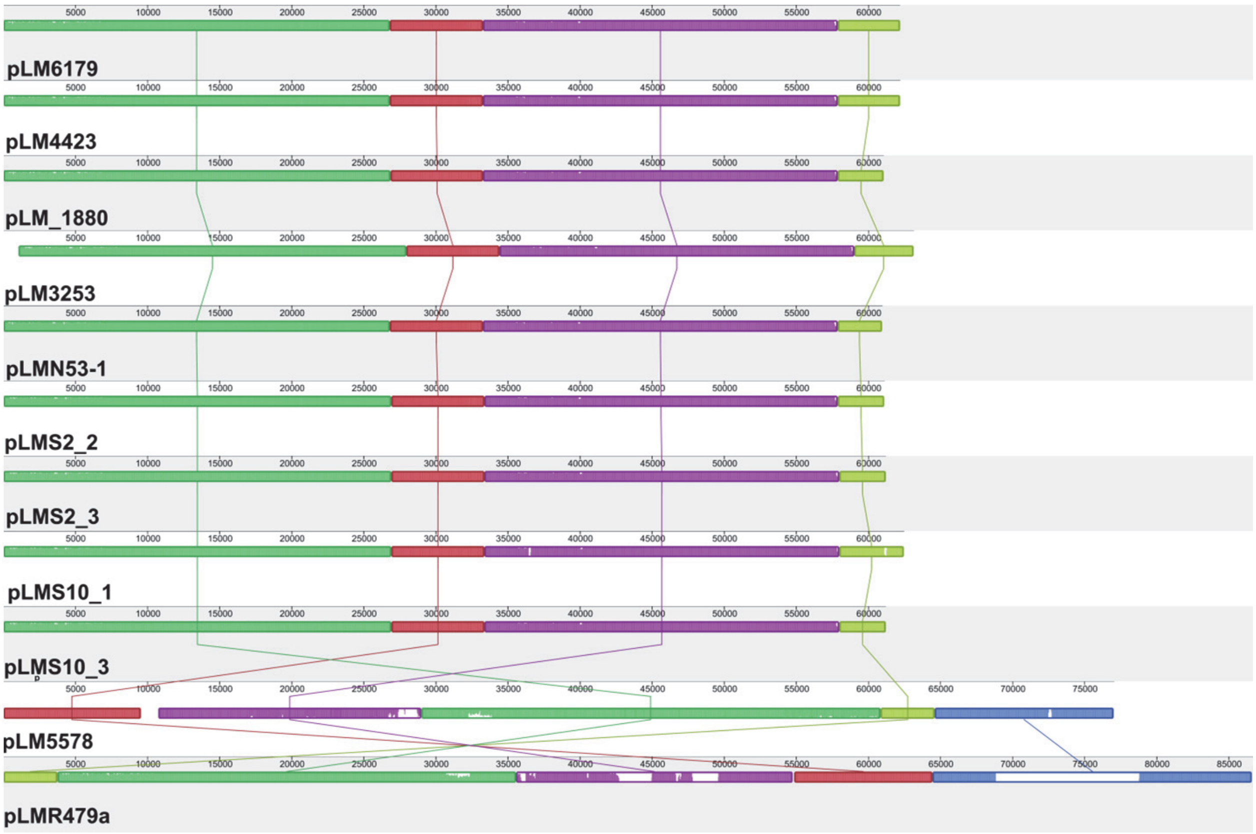

Supplementary Figure 5: Alignment of *L. monocytogenes* ST121 plasmids. The plasmids pLM6179, pLM4423, pLMN53-1, pLM3253, pLM_1880, pLMS2_2, pLMS2_3, pLMS10_1 and pLMS10_3 were aligned using Mauve (Darling, A.E., et al. (2010) PLoS One 5, e11147) and compared with related *L. monocytogenes* plasmids pLM5578 (ST120) (Gilmour et al. (2010) BMC Genomics. 2010, 11:120) and pLMR479a (ST8). Homologous regions are shown in the same color. The height of the similarity profile within each block corresponds to the average level of conservation in that region of the plasmids.
