# Supplementary material for: Genomes of sequence type 121 Listeria monocytogenes strains harbor highly conserved plasmids and prophages
Source: Front Microbiol. 2015 Apr 28;6:380. doi: 10.3389/fmicb.2015.00380 (PMC4412001; doi:10.3389/fmicb.2015.00380)
Supplement: Supplementary file 6 [file Image3.PDF]

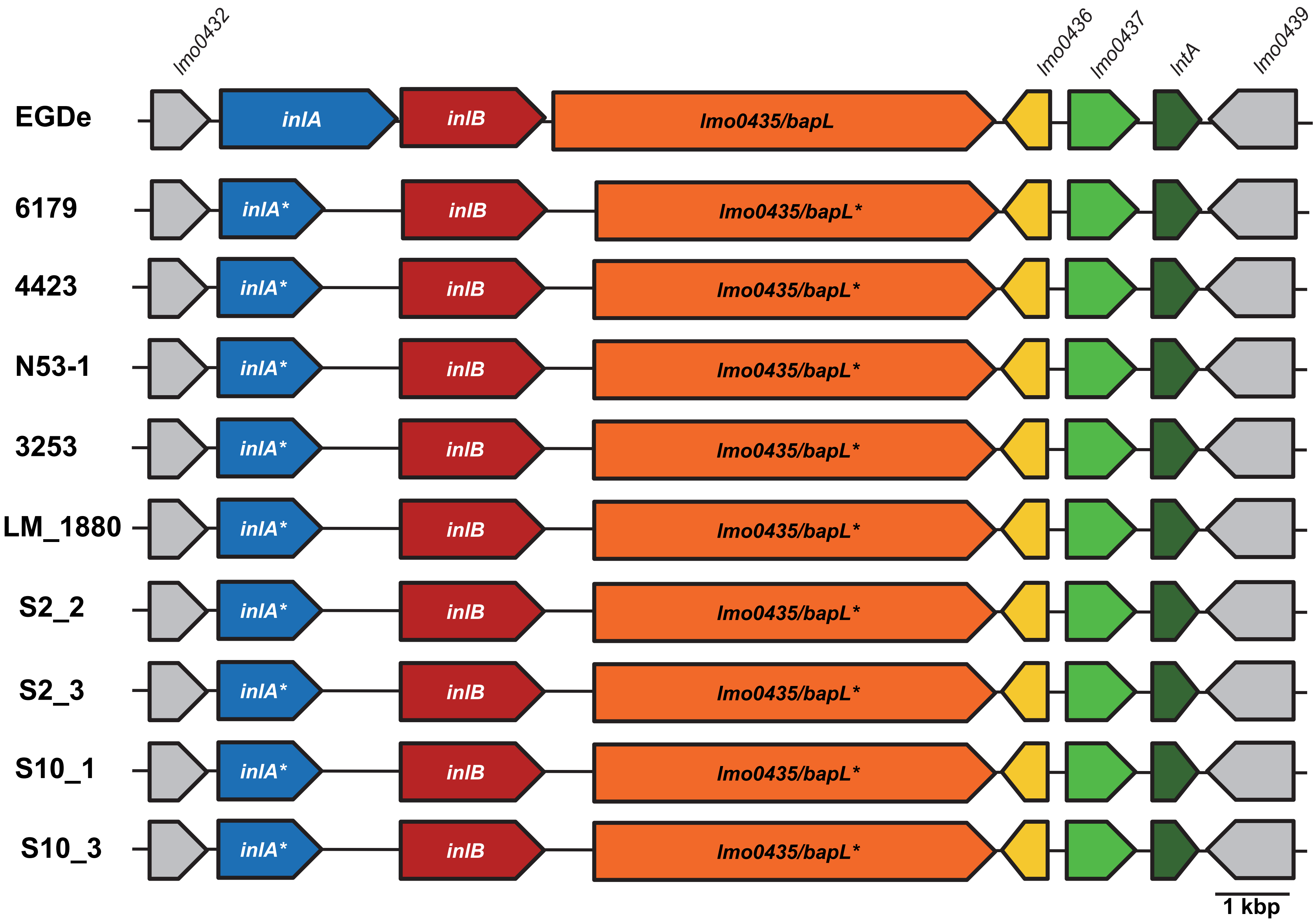

Supplementary Figure 3. Genomic organization of internalin AB loci (hypervariable hotspot 6) in ST121 *L. monocytogenes* strains. Homologous genes are shown in the same color. An asterisk indicates truncated genes.
